# Comparison of 20-Year Obesity-Associated Cancer Mortality Trends With Heart Disease Mortality Trends in the US

**DOI:** 10.1001/jamanetworkopen.2021.8356

**Published:** 2021-05-10

**Authors:** Christy L. Avery, Annie Green Howard, Hazel B. Nichols

**Affiliations:** 1 Department of Epidemiology, University of North Carolina at Chapel Hill, Chapel Hill; 2 Carolina Population Center, University of North Carolina at Chapel Hill, Chapel Hill; 3 Department of Biostatistics, University of North Carolina at Chapel Hill, Chapel Hill

## Abstract

**Importance:**

Heart disease and cancer are the 2 major diseases associated with mortality risk in the United States. Four decades of improvements in heart disease mortality slowed after 2011; this slowing has been associated with the obesity epidemic. The same pattern has not been observed for total cancer mortality. However, trends in total cancer mortality may obscure patterns specific to obesity-associated cancers.

**Objective:**

To investigate whether trends in obesity-associated cancer mortality mirror the slowed mortality improvements observed for heart disease associated with the obesity epidemic.

**Design, Setting, and Participants:**

This cross-sectional study compared US mortality trends for *International Statistical Classification of Diseases and Related Health Problems, Tenth Revision*–defined cancer (total cancer, obesity-associated cancer, and cancer not associated with obesity) and heart disease deaths from January 1, 1999, to December 31, 2018. Data were included on decedents with complete information on the underlying cause of death, age, sex, race, and ethnicity.

**Exposures:**

Changes in age-adjusted cause-specific mortality rates between 1999-2011 and 2011-2018 were compared.

**Main Outcomes and Measures:**

Annual relative rates of change in age-adjusted mortality rates (AAMRs) in the overall population and stratified by sex, race, and ethnicity were estimated using Poisson regression. Differences in AAMR annual relative rates of change before and after 2011 were evaluated using Wald tests.

**Results:**

A total of 50 163 483 decedents met the inclusion criteria (50.1% female decedents, 79.9% non-Hispanic White decedents, and 11.7% non-Hispanic Black decedents; mean [SD] age, 72.8 [18.5] years). In contrast with heart disease mortality, for which improvements slowed between 1999-2011 and 2011-2018, decreases in total cancer AAMR relative change accelerated between 1999-2011 (−1.48 [95% CI, −1.43 to −1.52]) and 2011-2018 (−1.77 [95% CI, −1.67 to −1.86]) (*P* < .001). For obesity-associated cancer mortality, which accounted for approximately 33% of total cancer deaths annually, decreases in annual AAMR relative change decelerated from −1.19 (95% CI, −1.13 to −1.26) in 1999-2011 to −0.83 (95% CI, −0.70 to −0.96) in 2011-2018 (*P* < .001). The largest decelerations in obesity-associated cancer mortality were observed for female decedents (–1.45 [95% CI, –1.36 to –1.53] in 1999-2011 and –0.91 [95% CI, –0.75 to –1.07] in 2011-2018; *P* < .001) and non-Hispanic White individuals (–1.16 [95% CI, –1.09 to –1.22] in 1999-2011 and –0.68 [95% CI, –0.55 to –0.81] in 2011-2018; *P* < .001).

**Conclusions and Relevance:**

Slowing improvements in obesity-associated cancer mortality were obscured when considering total cancer mortality. These findings potentially signal a changing profile of cancer-associated mortality that may parallel trends previously observed for heart disease as the consequences of the obesity epidemic are understood.

## Key Points

**Question:**

Did US mortality trends during 1999-2011 and 2011-2018 differ for obesity-associated cancers and cancers not associated with obesity?

**Findings:**

Using 20 years of cross-sectional mortality data, this study found that after 2011, mortality improvements accelerated for cancers not associated with obesity. In contrast, mortality improvements decelerated for obesity-associated cancers, paralleling recent trends for heart disease mortality.

**Meaning:**

The study results are consistent with the hypothesis that the obesity epidemic may have been associated with slowed improvement in obesity-associated cancer mortality.

## Teaser

This cross-sectional study investigates whether trends in obesity-associated cancer mortality mirror the slowed mortality improvements observed for heart disease associated with the obesity epidemic.

## Introduction

Cancer and heart disease share common risk factors^1^ and are the 2 major diseases associated with US mortality.^2^ After 4 decades of decline, annual improvements in heart disease mortality slowed in 2011; this slowing has been associated with the obesity epidemic.^3^ During the past 50 years, the prevalence of obesity has tripled,^4^ with approximately 2 in 5 US adults now being classified as obese.^5^ In addition to fueling a public health crisis with financial costs projected to double every decade to approximately $900 billion by 2030,^6,7^ obesity also has the potential to reverse gains in life expectancy achieved during the past century,^8^ with detrimental effects on par with cigarette smoking.^9^ Despite carcinogenic changes induced by obesity,^10^ a parallel deceleration in total cancer mortality rates during the same period has not been observed.^11^ However, not all cancer types have been linked to obesity. Therefore, we compared 20-year US mortality trends for cancer and heart disease, including both total cancer and separating cancer deaths for obesity-associated cancers and cancers not associated with obesity.

## Methods

Death information (counts, underlying causes, population size estimates, and demographic data) from January 1, 1999, to December 31, 2018, were retrieved from the Centers for Disease Control and Prevention online Wide-Ranging Online Data for Epidemiologic Research (WONDER) underlying cause of death database for US residents (50 states and the District of Columbia).^12^ Decedents missing information on cause of death (n = 264 849), age (n = 4507), sex (n = 0), race (n = 0), or ethnicity (n = 139 937) were excluded, leaving 50 163 483 deaths across 20 years. The institutional review board of the University of North Carolina at Chapel Hill determined that this study was exempt from review based on the use of publicly available data. This study followed the reporting requirements of the Strengthening the Reporting of Observational Studies in Epidemiology (STROBE) reporting guideline for cross-sectional studies.^13^

Underlying cause of death was assigned based on *International Statistical Classification of Diseases and Related Health Problems, Tenth Edition* codes as heart disease (codes I00-I09, I11, I13, and I20-I51) or cancer (malignant neoplasms [codes C00-C97]). Cancer was further examined as obesity-associated cancer (adenocarcinoma of the esophagus [code C15], upper stomach [code C16.0], colon and rectum [codes C18 and C20], liver [code C22], gallbladder [code C23], pancreas [code C25], postmenopausal breast [code C50], uterine [code C54], ovarian [code C56], kidney [code C64], meningioma [code C70], thyroid [code C73], and multiple myeloma [code C90.0]) and cancer not associated with obesity (defined as all remaining malignant neoplasms).^14^ Postmenopausal breast cancer and premenopausal breast cancer are not distinguished by *International Statistical Classification of Diseases and Related Health Problems, Tenth Edition* codes; therefore, we used 50 years of age as a proxy for menopausal status and evaluated the sensitivity of our results to this threshold by alternatively defining postmenopausal status at the ages of 45 years and 55 years. No other sensitivity analyses were performed.

To enable comparisons with the published literature as well as the examination of subgroup-specific effects and age-adjusted mortality rates (AAMRs) in the overall population and stratified by sex (female or male), the 4 available racial categories (non-Hispanic American Indian or Alaska Native, non-Hispanic Asian or Pacific Islander, non-Hispanic Black or African American, and non-Hispanic White), and ethnicity (Hispanic) were calculated using the direct method with the 2000 US Census as the standard population.^15^ Annual relative rates of change in AAMRs were estimated using Poisson regression, allowing for overdispersion and including a piecewise linear spline with 1 knot at 2011. These modeling decisions were informed by prior research that identified an inflection point in 2011 for US heart disease mortality,^16^ the limited number of data points (20), and the need for a common analytic strategy that enabled comparisons of cancer and heart disease mortality trends in the overall population and when stratified by sex, race, ethnicity, and obesity-associated cancer and cancer not associated with obesity. Differences in AAMR annual relative rates of change before and after 2011 were evaluated using Wald tests. All *P* values were from 2-sided tests and results were deemed statistically significant at *P* < .05.

## Results

A total of 50 163 483 decedents met the inclusion criteria (50.1% female decedents, 79.9% non-Hispanic White decedents, and 11.7% non-Hispanic Black decedents; mean [SD] age, 72.8 [18.5] years). Cancer or heart disease accounted for 53.6% of total deaths in 1999 and 44.4% of total deaths in 2018. Decreases in AAMRs were observed for both cancer and heart disease but showed different patterns of change. As previously reported, heart disease mortality improvements slowed between 1999-2011 (annual AAMR relative change, −3.80 [95% CI −3.66 to −3.93]) and 2011-2018 (annual AAMR relative change, −0.72 [95% CI, −0.45 to −0.99]) (Table).^11^ Conversely, total cancer mortality improvements accelerated between 1999-2011 (annual AAMR relative change, −1.48 [95% CI, −1.43 to −1.52]) and 2011-2018 (annual AAMR relative change, −1.77 [95% CI, −1.67 to −1.86]).

**Table.** Age-Adjusted Heart Disease and Cancer Mortality Rates and Annual Relative Rates of Change in the United States, 1999-2018

Disease	AAMR^a^			AAMR annual relative rate of change, estimate (95% CI)^b^		
	1999	2011	2018	1999-2011	2011-2018	*P* value for Wald test of difference before and after 2011
**Heart disease**						
Overall	265.5	173.3	163.1	−3.80 (−3.66 to −3.93)	−0.72 (−0.45 to −0.99)	<.001
Male	329.7	217.5	206.7	−3.68 (−3.54 to −3.81)	−0.54 (−0.27 to −0.80)	<.001
Female	217.4	138.5	127.5	−4.05 (−3.88 to −4.21)	−1.07 (−0.74 to −1.40)	<.001
Non-Hispanic						
White	263.8	175.6	167.5	−3.65 (−3.51 to −3.79)	−0.40 (−0.12 to −0.68)	<.001
Black	337.4	219.3	209.3	−3.85 (−3.63 to −4.07)	−1.01 (−0.57 to −1.46)	<.001
Asian or Pacific Islander	156.5	93.8	85.1	−4.15 (−3.91 to −4.39)	−1.81 (−1.32 to −2.31)	<.001
American Indian or Alaskan Native	216.5	161.0	145.8	−2.41 (−2.05 to −2.78)	−0.95 (−0.23 to −1.68)	.004
Hispanic	205.8	123.9	112.3	−4.38 (−4.11 to −4.65)	−1.77 (−1.22 to −2.33)	<.001
**All cancer**						
Overall	200.3	168.7	148.8	−1.48 (−1.43 to −1.52)	−1.77 (−1.67 to −1.86)	<.001
Male	251.3	203.7	176.3	−1.78 (−1.73 to −1.83)	−2.03 (−1.93 to −2.14)	<.001
Female	167.2	143.8	128.4	−1.33 (−1.28 to −1.39)	−1.60 (−1.49 to −1.72)	<.001
Non-Hispanic						
White	201.3	173.0	154.4	−1.30 (−1.25 to −1.35)	−1.59 (−1.49 to −1.69)	<.001
Black	255.9	204.0	173.8	−1.98 (−1.91 to −2.05)	−2.19 (−2.05 to −2.33)	.03
Asian or Pacific Islander	123.4	106.7	93.4	−1.19 (−1.03 to −1.35)	−1.73 (−1.42 to −2.05)	.01
American Indian or Alaskan Native	147.7	141.2	128.1	−0.11 (0.27 to −0.49)	−2.12 (−1.38 to −2.87)	<.001
Hispanic	134.8	117.1	107.3	−1.24 (−1.14 to −1.34)	−1.32 (−1.12 to −1.52)	.56
**Cancers not associated with obesity**						
Overall	133.8	110.3	93.8	−1.62 (−1.57 to −1.67)	−2.29 (−2.19 to −2.39)	<.001
Male	188.7	146.6	122.5	−2.12 (−2.07 to −2.17)	−2.59 (−2.50 to −2.68)	<.001
Female	98.1	84.6	72.9	−1.25 (−1.18 to −1.33)	−2.10 (−1.96 to −2.25)	<.001
Non-Hispanic						
White	135.5	115.0	99.3	−1.38 (−1.32 to −1.43)	−2.07 (−1.96 to −2.18)	<.001
Black	167.2	127.2	104.1	−2.34 (−2.27 to −2.42)	−2.78 (−2.62 to −2.94)	<.001
Asian or Pacific Islander	78.1	63.5	54.9	−1.60 (−1.38 to −1.83)	−1.83 (−1.39 to −2.28)	.46
American Indian or Alaskan Native	94.4	88.7	75.9	−0.31 (0.09 to −0.72)	−3.00 (−2.18 to −3.83)	<.001
Hispanic	83.7	68.1	60.4	−1.72 (−1.60 to −1.85)	−1.75 (−1.50 to −2.01)	.87
**Obesity-associated cancers**						
Overall	66.5	58.4	54.9	−1.19 (−1.13 to −1.26)	−0.83 (−0.70 to −0.96)	<.001
Male	62.6	57.0	53.8	−0.83 (−0.74 to −0.93)	−0.71 (−0.53 to −0.88)	.31
Female	69.1	59.1	55.5	−1.45 (−1.36 to −1.53)	−0.91 (−0.75 to −1.07)	<.001
Non-Hispanic						
White	65.7	58.0	55.1	−1.16 (−1.09 to −1.22)	−0.68 (−0.55 to −0.81)	<.001
Black	88.7	76.8	69.7	−1.34 (−1.24 to −1.43)	−1.28 (−1.09 to −1.46)	.63
Asian or Pacific Islander	45.3	43.2	38.5	−0.54 (−0.37 to −0.71)	−1.60 (−1.27 to −1.93)	<.001
American Indian or Alaskan Native	53.3	52.6	52.3	0.25 (0.75 to −0.26)	−0.77 (0.17 to −1.72)	.13
Hispanic	51.2	49.0	47.0	−0.52 (−0.39 to −0.65)	−0.75 (−0.51 to −1.00)	.18

Abbreviation: AAMR, age-adjusted mortality rate.

^a^ Per 100 000 persons.

^b^ Annual relative rate of change and Wald test were estimated from Poisson models assuming a linear trend and a piecewise linear spline at 2011.

Cancers not associated with obesity accounted for 66.8% of deaths for which cancer was the underlying cause of death in 1999, decreasing to 62.6% in 2018. For cancers not associated with obesity, decreases in mortality accelerated between 1999-2011 (annual AAMR relative change, −1.62 [95% CI, –1.57 to −1.67]) and 2011-2018 (annual AAMR relative change, −2.29 [95% CI, −2.19 to −2.39]; *P* < .001 for difference in AAMR) (Table). In contrast, decreases in obesity-associated cancer mortality slowed after 2011 (annual AAMR relative change: −1.19 [95% CI, −1.13 to −1.26] in 1999-2011 vs −0.83 [95% CI, −0.70 to −0.96] in 2011-2018) (*P* < .001 for difference in AAMR). Female decedents and non-Hispanic White individuals had the largest annual decreases in AAMRs for cancer not associated with obesity in 2011-2018 compared with 1999-2011 (female decedents: –1.25 [95% CI, –1.18 to –1.33] in 1999-2011 and –2.10 [95% CI, –1.96 to –2.25] in 2011-2018 [*P* < .001]; non-Hispanic White decedents: –1.38 [95% CI, –1.32 to –1.43] in 1999-2011 and –2.07 [95% CI, –1.96 to –2.18] in 2011-2018 [*P* < .001]), as well as the greatest slowing in obesity-associated cancer annual AAMRs in 2011-2018 compared with 1999-2011 (female decedents: –1.45 [95% CI, –1.36 to –1.53] in 1999-2011 and –0.91 [95% CI, –0.75 to –1.07] in 2011-2018 [*P* < .001]; non-Hispanic White decedents: –1.16 [95% CI, –1.09 to –1.22] in 1999-2011 and –0.68 [95% CI, –0.55 to –0.81] in 2011-2018 [*P* < .001]) (Table, Figure). Results were consistent in sensitivity analyses that modified the age threshold for postmenopausal breast cancer mortality from 55 years to 45 years.

**Figure.**
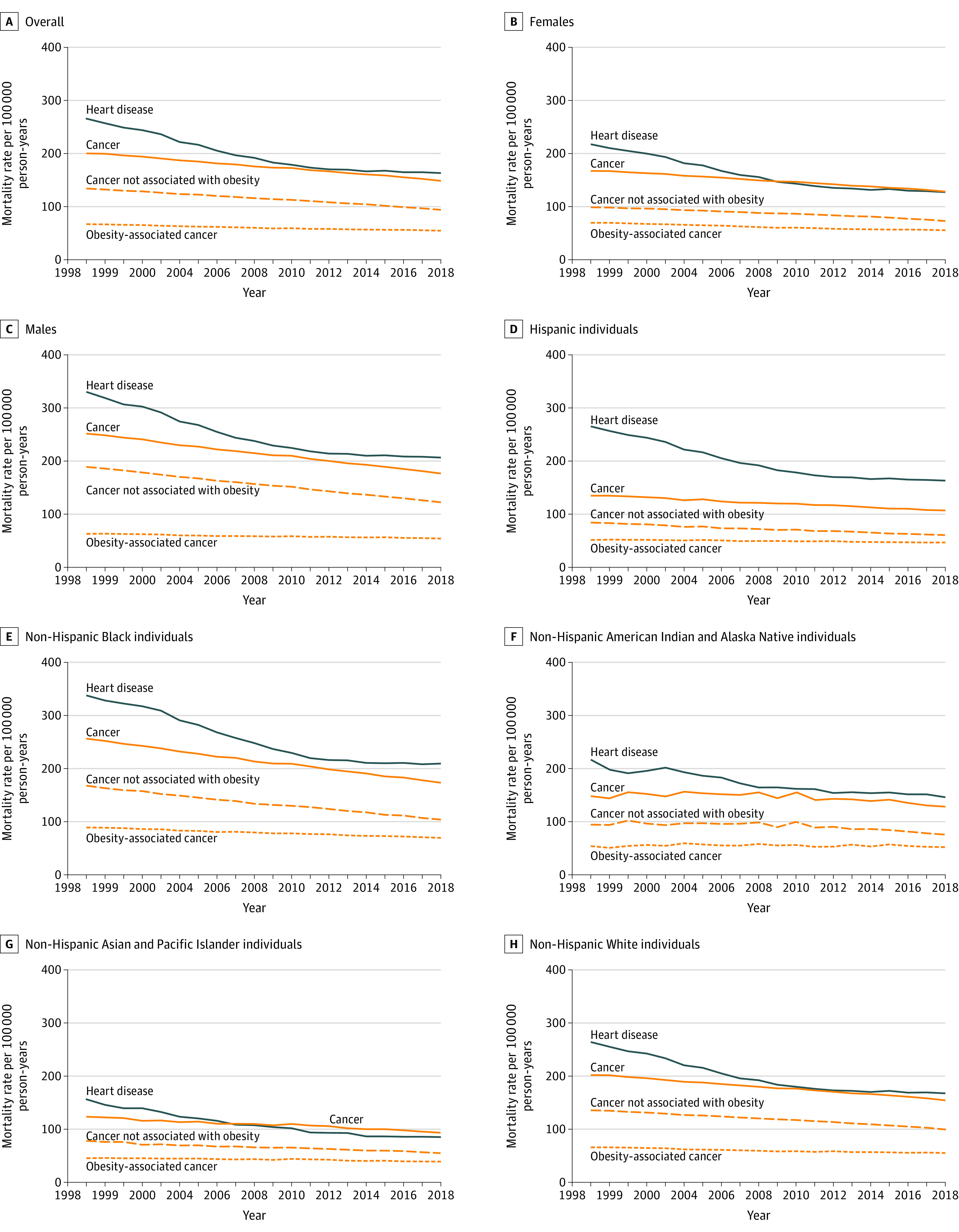
Age-Standardized Heart Disease and Cancer Annual Mortality Rates in the Overall Population and by Sex, Race, and Ethnicity in the United States, 1999-2018

## Discussion

By integrating 20 years of cancer mortality data, we demonstrated that trends in obesity-associated cancer mortality showed signs of recent deceleration, consistent with recent findings for heart disease mortality.^11^ These findings were obscured when considering total cancer mortality and potentially signal a changing profile of obesity-associated cancer mortality as the consequences of the obesity epidemic are understood.

Recent reports have described the overall decrease in cancer mortality^17,18,19^ but cautioned that cancer mortality rates have been stable or increasing from 2013 to 2017 for 8 of 19 of the most common cancer types among men and 6 of 20 of the most common cancer types among women.^17^ The largest mortality decreases were observed for melanoma of the skin and lung cancer, 2 cancers not associated with obesity.^17^ For obesity-associated cancers, stable or increasing mortality rates have been observed for liver and pancreatic cancer among both men and women as well as for uterine cancer among women.^17^ Mortality trends aggregating obesity-associated cancers and cancers not associated with obesity were not presented.

Our findings suggest that trends in obesity-associated cancer mortality and heart disease mortality showed slowing improvements that emerged in the second decade of the 21st century, although the demographic patterns underlying these trends differed. For heart disease, evidence of a deceleration was consistent across sex, race, and ethnicity. In contrast, decelerations in obesity-associated cancer mortality were more pronounced for female decedents and non-Hispanic White individuals. For Non-Hispanic Asian individuals and Pacific Islander individuals, an opposite pattern was observed, whereby decreases in obesity-associated cancer mortality accelerated. These findings are challenging to interpret because the prevalence of obesity has increased during the same time for both US women and US men as well as for most racial and ethnic groups.^5^ Longstanding disparities in obesity also exist, particularly among Hispanic and non-Hispanic Native Hawaiian and Black populations. Longitudinal data in diverse populations may help to more precisely define how obesity may have been associated with decelerations in both obesity-associated cancer mortality and heart disease mortality, including the role of longstanding disparities and the impact of recent trends whereby obesity occurs earlier in the life course and is of greater severity. Extending these efforts to include other diseases associated with obesity also could help strengthen inference and provide a more integrated picture of the degree to which US mortality trends are associated with obesity.

### Strengths and Limitations

Despite several strengths, including the use of contemporary national mortality and population data, there are important limitations to this study. First, we restricted our attention to national cause of death data and population counts, from which we estimated cause-specific mortality rates. Mortality rates represent the principal standardized source of health-related data at national, state, and local levels, enabling comparisons of different causes of death in large numbers of decedents to inform health policy development and to prevent or reduce premature mortality. However, there are limitations inherent in the use of mortality data to examine potential associations of obesity with health outcomes or concomitant trends in energy balance behaviors of physical inactivity and dietary patterns.^17,18^ As an established chronic disease risk factor, secular changes in obesity may be accompanied by improvements in early detection and treatment; for cancer, this scenario could increase cancer incidence while decreasing mortality for some cancer types. We attempted to address these challenges in part by triangulating evidence across different diseases,^20^ although we acknowledge that studies with measured body mass index preceding disease onset are needed to help clarify the association of obesity with both incidence and mortality. Second, we relied on unvalidated death certificate data. For cancer mortality, validation studies have suggested good to excellent reliability,^21^ which is in contrast to studies reporting overestimates of heart disease mortality when death certificates are the only source of information.^22^ Although these differences may affect comparisons between cancer and heart disease, the extent to which they are associated with trends in cancer overall or when examined by obesity subtype remains unclear. Third, we were limited in the degree to which we could examine heterogeneity by race because only 4 racial categories were included on the Centers for Disease Control and Prevention WONDER database: American Indian or Alaskan Native, Asian or Pacific Islander, Black or African American, and White. This racial categorization is inconsistent with a 1997 Office of Management and Budget directive^23^ that separated the Asian or Pacific Islander racial category into 2 categories: (1) Asian and (2) Native Hawaiian and other Pacific Islander. Native Hawaiian individuals and other Pacific Islander individuals are more than 4 times as likely as Asian individuals to be obese.^24^ Disparities in heart disease and cancer incidence and mortality also have been reported for Native Hawaiian individuals,^25^ motivating future work that evaluates these populations separately.

## Conclusions

The negative associations of obesity with overall life expectancy were first reported by Olshansky et al in 2005.^9^ In their report, Olshansky et al^9^ speculated that, in the absence of successful interventions, the largest impact of obesity was likely to emerge in the first half of the 21st century. The findings of our study suggest that heart disease and obesity-associated cancer mortality rates have continued to improve, but at a slowing pace. Whether the findings of decelerating mortality rates potentially signal a changing profile of cancer and heart disease mortality as the consequences of the obesity epidemic are realized remains to be seen.
